# Microbial volatile organic compounds and olfactory receptors in wound malodor

**DOI:** 10.3389/fcimb.2026.1833939

**Published:** 2026-07-10

**Authors:** Sophie Charlotte Liegenfeld, Ewa Klara Stuermer, Nele Pelzer, Clara Nathrath, Nicolas Krueger, Markus Geissen, Mandy Dittmer

**Affiliations:** Translational Wound Research, Department of Vascular Medicine, University Medical Center Hamburg-Eppendorf (UKE), Hamburg, Germany

**Keywords:** chronic wounds, olfactory receptors, volatile organic compounds, wound malodor, wound microbiome

## Abstract

Wound odor is a common symptom in patients with chronic wounds. Malodorous wounds can result in embarrassment, anxiety, and, therefore, in social isolation of the patient. Current treatment options are limited and focused on masking the odor. Current approaches to wound odor management have notable limitations and fail to adequately address existing therapeutic needs. Wound odor is multifactorial, with a major contribution from volatile organic compounds (VOCs) emitted by microorganisms and necrotic tissue. Several VOCs have been identified as key drivers of malodor, such as short-chain fatty acids, dimethyl-trisulfide, specific alcohols, ketones, and aldehydes. These compounds are detected by the human olfactory system, including olfactory receptors (ORs) expressed on olfactory sensory neurons (OSNs). The human olfactory system comprises more than 400 ORs, which enable the detection of a vast array of odorants, however only a small fraction of these receptors has been deorphanized. This systematic review will evaluate therapeutic clinical strategies addressing malodor and compile current knowledge on VOCs that cause wound odor and the ORs associated with their detection. It summarizes the existing evidence on deorphanized ORs that detect these VOCs in malodorous wounds and outlines experimental mechanisms of deorphanization. Identifying wound-odor-associated VOCs and their corresponding ORs provides a framework for novel therapeutic strategies, such as antagonizing the OR to suppress odor perception. This may directly improve the quality of life of patients with chronic malodorous wounds, particularly in malignant wounds and palliative care.

## 1 Introduction

Chronic wounds represent a global health challenge and a socio-economic burden as it affects around 1.5 to 2 million people in Europe (Lindholm and Searle, 2016). Within the clinical manifestations, wound odor is cited as one of the most distressful aspects, resulting in embarrassment, anxiety, and social isolation of the patient (Gethin et al., 2023). The odor is a burden not only on the patient but also on family members and health care professionals (HCPs). Current strategies against wound odor focus mainly on masking the malodor using non-standardized home remedies or off-label pharmaceuticals or reducing the bacterial burden through antimicrobial interventions (Gethin et al., 2014; Samala and Davis, 2015).

To move beyond symptomatic management and enable mechanism-based interventions, it is essential to understand the biochemical origin of wound odor and its perception. At the molecular level, wound malodor is primarily driven by volatile organic compounds (VOCs) emitted from the wound environment. These VOCs, e.g., short-chain fatty acids, ketones, alcohols, and aldehydes, primarily arise from interrelated factors, such as bacterial colonization and its metabolic byproducts, necrotic tissue, and local hypoxia (Hara et al., 2020; Stuermer et al., 2025). Different bacterial strains emit characteristic VOCs, enabling their identification based on odor signatures, with analytical technologies still undergoing refinement (Ramírez-Guízar et al., 2017). *Pseudomonas aeruginosa* (*P. aeruginosa*), one of the main causative agents for wound infections, is often associated with strong malodor (Salinas Alvarez et al., 2019). It emits a sulfur-containing VOC, dimethyl-trisulfide (DMTS), which is described as smelling “sulphury and onion-like” (Shirasu et al., 2009). Wound odor is ultimately perceived because these VOCs interact with olfactory receptors (ORs) in the nasal cavity, which translate chemical signals into sensory experience.

Humans have about 380–400 different functional ORs, which belong to the largest G protein-coupled receptor (GPCR) family (Odoemelam et al., 2025). Each OR has the ability to bind a large diversity of odorant molecules, so-called ligands, with diverse physicochemical properties. OR-odorant interactions are complex and sometimes promiscuous, with individual receptors responding to multiple odorants and single odorants activating multiple receptors (Launay et al., 2012; Achebouche et al., 2022).

Despite considerable progress in receptor deorphanization, a substantial proportion of the approximately 400 functional human olfactory receptors remain orphan receptors, meaning that no cognate odorant has yet been conclusively identified (Civelli et al., 2013; Jobe and Vijayan, 2024). The process of identifying odorants that activate a receptor is referred to as receptor deorphanization (Civelli et al., 2013), and enables the systematic assignment of specific microbial or tissue-derived VOCs to defined human receptors. Several deorphanization strategies have been described in literature and are commonly grouped by biological context (Zhuang and Matsunami, 2008; Bavan et al., 2014). The most widely used strategy to determine VOC-OR binding is heterologous expression in human cell lines, in which human ORs are expressed in suitable cell lines such as HEK293T, Hana3, and HeLa (Peterlin et al., 2014; Ieki et al., 2022). An alternative *in vitro* approach relies on the direct stimulation of primary olfactory sensory neurons (OSNs) with odorants either *in situ* or in culture, retaining their physiological receptor expression and signaling machinery (Peterlin et al., 2014). However, OSN deorphanization is largely restricted to rodent models, is technically demanding and inherently low-throughput, which limits its suitability for large-scale deorphanization efforts. Beyond cell-based assays, *in silico* and genomics-based approaches aim to identify OR-odorant relationships from molecular features and human perceptual data, providing a predictive framework to guide targeted deorphanization efforts. *In silico* prediction relies on computational modelling of OR structures and their ligand-binding pockets to identify candidate odorants and generate potential OR-odorant matches (Audouze et al., 2014; Kim and Goddard, 2014; Kowalewski and Ray, 2020; Oh, 2021; Berwal et al., 2025).

In practice, the different pipelines are complementary rather than independent. Such mappings may also inform the development of diagnostic detection systems, including electronic noses and receptor-based biosensors. Antagonization, on the other hand, describes the process by which one odorant binds to an OR but does not fully, or not at all, activate it, thereby blocking another more potent odorant from binding and inducing a full signal. With an antagonist targeting a wound-associated VOC, it may be possible to attenuate or remove malodor at the level of olfactory perception rather than masking it (Stuermer et al., 2025), as already described for Timberol^®^ in the case of fish odors (Wallrabenstein et al., 2013; Wallrabenstein et al., 2015).

**Figure 1:**
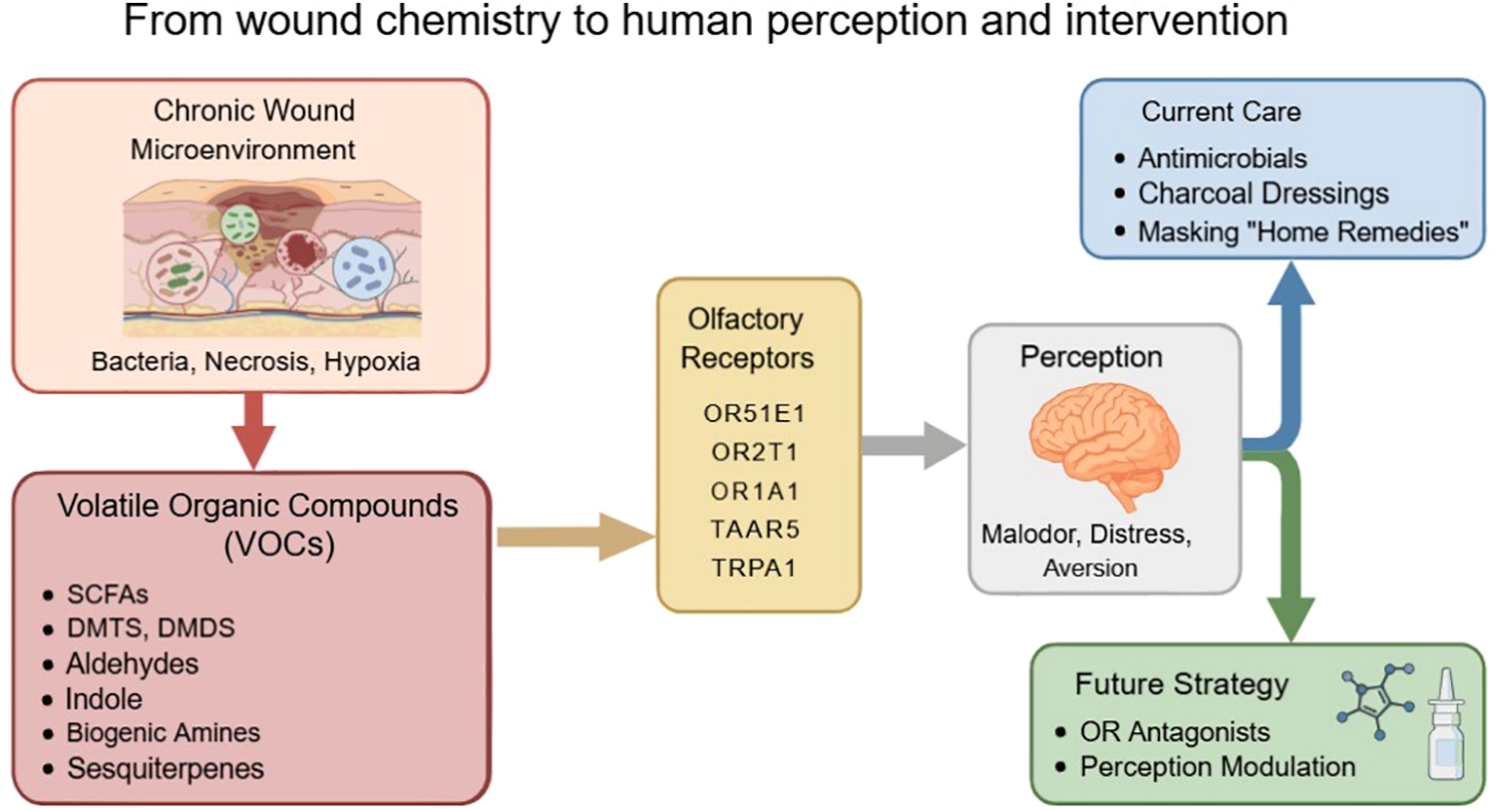
From wound chemistry to human perception and intervention. Schematic overview of how wound-derived VOCs are generated, detected by human olfactory receptors, and how current treatments differ from future receptor-based strategies targeting odor perception.

In this review, the VOCs responsible for wound odor and their corresponding olfactory receptors will be described. Recent advances in deorphanizing these receptors will be outlined, including the *in vitro* methods used, and how antagonizing specific receptors might reduce odor perception. Translational implications of these insights for developing new diagnostic and therapeutic strategies are also addressed. A conceptual overview of this pathway, from the wound environment through VOC emission, olfactory detection, and contrasting current versus future therapeutic approaches, is summarized in **Figure 1**.

## 2 Materials and methods

**Figure 2:**
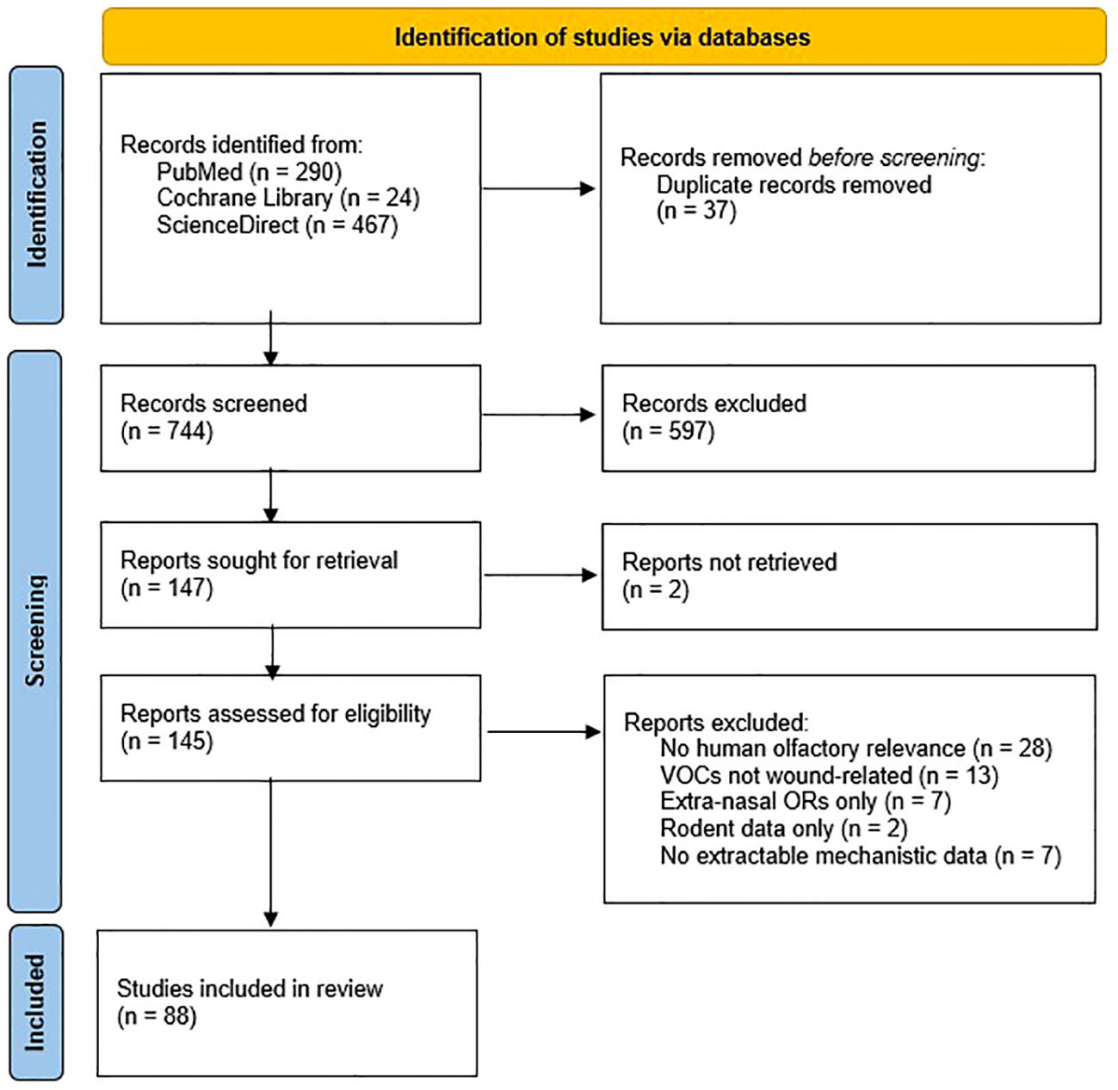
PRISMA 2020 flow diagram of study selection. Overview of database search, screening, eligibility assessment, and final inclusion of studies in this systematic review, resulting in 88 included publications.

This systematic review was conducted and reported in accordance with the Preferred Reporting Items for Systematic Reviews and Meta-Analyses (PRISMA) 2020 statement. The study selection process is summarized in the PRISMA 2020 flow diagram (**Figure 2**).

### 2.1 Literature search strategy

A systematic literature review was conducted using the electronic databases MEDLINE (via PubMed), ScienceDirect, and the Cochrane Library. Publications between January 2000 to December 2025 were considered. The final database search was conducted in February 2026. The following keywords were included: “bacterial volatile organic compounds”; “volatile organic compounds” AND “wound”; “wound odor”; “malodorous wounds”; “human olfactory receptors”; “deorphanization” AND “olfactory receptors” and “bacterial volatile organic compounds”. Due to the full-text nature of ScienceDirect searches and the high number of non-relevant records retrieved, database-specific search adaptations were applied to improve precision, including the use of exclusion operators (e.g., NOT plants; NOT food) to eliminate non-relevant disciplinary contexts. In ScienceDirect, searches were further restricted to research articles and review articles. The complete database-specific search strategies are provided in **Supplementary Table 1**. Overall, 290 articles were identified via PubMed, 24 via Cochrane Library, and 467 via ScienceDirect. After removing 37 duplicate records, 744 unique records remained for further analysis (**Figure 2**).

### 2.2 Inclusion and exclusion criteria

Only peer-reviewed articles published in English or German were considered. Studies were included if they investigated VOCs associated with wounds or bacterial metabolism, identified odors responsible for malodor in wounds and infection, or examined human olfactory receptors involved in the detection of such compounds. In addition, studies describing current clinical strategies for managing wound odor, including topical, antimicrobial, and adsorptive approaches, were considered to provide clinical context. Studies were excluded if they addressed ORs located at other sites than the nasal cavity, as extra-nasal ORs are not involved in odor perception and therefore fall outside of the scope of this review.

### 2.3 Review protocol and study selection

A prospectively registered review protocol was not available for this study. However, the review question, eligibility criteria, search strategy, and study selection procedures were predefined prior to commencement of the literature search and were applied consistently throughout the review process. Data extraction was performed independently by two reviewers and discrepancies were resolved through discussion and consensus. Following duplicate removal, titles and abstracts were independently screened by two reviewers to exclude records that were clearly outside the scope of the review. Of the 744 unique records identified, 147 articles were considered potentially eligible and retrieved for full-text assessment. Full-text articles were subsequently evaluated according to the predefined inclusion and exclusion criteria (**Figure 2**). As was the case during the initial selection process, disagreements regarding study eligibility were resolved through discussion and consensus between the reviewers; unresolved disagreements were referred to a third reviewer for adjudication. Following full-text assessment, 88 publications met the eligibility criteria and were included in the qualitative synthesis. Due to the heterogeneous nature of the included evidence, encompassing clinical studies, microbiological investigations, receptor deorphanization studies, animal experiments, and computational modelling approaches, a formal risk-of-bias assessment using a single standardized instrument was not considered appropriate. Instead, the included studies were qualitatively assessed by reviewers with respect to study design, experimental methodology, and evidential strength, taking into account differences between clinical investigations, microbial VOC studies, receptor deorphanization experiments, animal models, and computational prediction approaches.

### 2.4 Data extraction and evidence synthesis

For each included study, relevant information was extracted regarding study characteristics, investigated microorganisms, volatile organic compounds (VOCs), wound type or experimental model, olfactory receptors (ORs), deorphanization methodology, receptor antagonists, and reported clinical or translational implications where applicable. Additional methodological details relevant to the respective study type, such as receptor screening approaches, computational prediction methods, or VOC detection techniques, were also recorded. Given the heterogeneous nature of the included literature, a quantitative synthesis was not feasible. Extracted data were therefore synthesized narratively and organized into thematic categories addressing (i) VOCs associated with wound malodor, (ii) current clinical strategies for odor management, (iii) deorphanization of olfactory receptors responsive to wound-associated VOCs, and (iv) translational opportunities for receptor antagonization and odor-targeted interventions. To facilitate interpretation of the available evidence, studies were considered according to their primary evidence type, including clinical wound studies, microbial VOC studies, human olfactory receptor studies, animal studies, and computational prediction studies.

## 3 Results

### 3.1 Spectrum of volatile organic compounds detected in chronic wounds

The odor of malodorous wounds is primarily driven by VOCs emitted by microorganisms and necrotic tissue. VOCs, which are commonly associated with an unpleasant odor in festering wounds, include dimethyl trisulfide (sulphur aroma) and short-chain fatty acids (SCFAs): acetic acid (sour aroma), isobutyric acid (cheese aroma), butyric acid (cheese and vomit aroma), and isovaleric acid (cheese and foot aroma) (Shirasu et al., 2009). Additional volatiles such as amino acids, ammonia, and hydrogen cyanide further contribute to the olfactory profile of chronic wounds (Azimzadeh et al., 2025). In clinical practice, certain odors are often intuitively associated with specific pathogens, reflecting the characteristic VOC profiles produced by individual bacterial species. Furthermore, specific VOCs could be identified and assigned to bacterial or fungal species.

A prominent example is *P. aeruginosa*, which is strongly linked to DMTS and its distinctive sulphurous odor, reflecting the sulfur-based anaerobic metabolism (**Table 1**) that is characteristic of persistent *P. aeruginosa* wound colonization (Shirasu et al., 2009). *Staphylococcus aureus* (*S. aureus*), another key pathogen in chronic wounds, produces a characteristic VOC signature, including isovaleric acid and 2-methyl-butanal, which contribute substantially to the malodor of infected wounds (Bos et al., 2013). As one of the five most prevalent bacterial genera in chronic wounds, *Corynebacterium* spp. is of particular interest because of its distinctive VOC profile, which includes a combination of isovaleric acid and 3-Methyl-2-hexenoic acid (3M2H/TMHA; rancid sweat-like aroma) and the sulphuric VOCs 3-methyl-3-sulfanylhexan-1-ol (3M3SH) (Starkenmann et al., 2005; Hara et al., 2014; Ashrafi et al., 2017; Liegenfeld et al., 2025). In the context of malignant wounds, *Proteus mirabilis* (*P. mirabilis*) and *Fusobacterium necrophorum* (*F. necrophorum*) are two major contributors to severe malodor (Thuleau et al., 2018). In isolated bacterial cultures, *P.* mirabilis is associated with 3-methylbutanal (fruity, cheesy aroma), dimethyl disulphide (DMDS; sulphurous, sweet-cheesy), DMTS, and indole (fecal, overripe sweet aroma), while *F. necrophorum* is characterized by the production of DMTS, phenol (medicinal, antiseptic-like), DMDS, and indole (Thuleau et al., 2018) (**Table 1**).

**Table 1:** Overview of the prevalent VOCs responsible for the malodor in chronic wounds and their smell description.

VOC	Chemical class	Typical odor description	Principal source(s) in wounds	Evidence type	Key references
Dimethyl trisulfide (DMTS)	Sulfur compound	Sulphurous, garlic-like	*Pseudomonas aeruginosa*, anaerobes	Wound-derived samples (malignant breast cancer wounds) and bacterial culture studies	(Shirasu et al., 2009; Thuleau et al., 2018)
Dimethyl disulfide (DMDS)	Sulfur compound	Pungent, sweet–cheesy, putrid	*Proteus mirabilis*, *Fusobacterium necrophorum*	Wound-derived samples (malignant breast cancer wounds) and bacterial culture studies	(Thuleau et al., 2018)
3-Methyl-3-sulfanylhexan-1-ol (3M3SH)	Sulfur alcohol	Sulphurous, sweaty	*Corynebacterium* spp.	Bacterial culture studies	(Starkenmann et al., 2005; Hara et al., 2014; Ashrafi et al., 2017)
Acetic acid	Short-chain fatty acid	Sour, vinegar-like	Mixed bacterial flora	Wound-derived samples (fungating cancer wounds)	(Shirasu et al., 2009)
Isobutyric acid	Short-chain fatty acid	Cheesy, sweaty, rancid	*Staphylococcus aureus*, mixed flora	Wound-derived samples (fungating cancer wounds)	(Shirasu et al., 2009)
Butyric acid	Short-chain fatty acid	Rancid butter, vomit-like	Anaerobes, necrotic tissue	Wound-derived samples (fungating cancer wounds)	(Shirasu et al., 2009)
Isovaleric acid	Short-chain fatty acid	Cheesy, sweaty, foot-like	*Staphylococcus aureus*, *Corynebacterium* spp.	Wound-derived samples (fungating cancer wounds) and bacterial culture studies	(Shirasu et al., 2009; Bos et al., 2013)
Propionic acid	Short-chain fatty acid	Rancid, sweaty, body-odour–like	Mixed flora, *Candida* spp.	Wound-derived samples and fungal culture studies	(Costa et al., 2020; Fitzgerald et al., 2022)
3-Methyl-2-hexenoic acid (TMHA, 3M2H)	Fatty acid derivative	Rancid sweat, body odour	*Corynebacterium* spp.	Bacterial culture studies, human axillary microbiome studies	(Starkenmann et al., 2005; Hara et al., 2014; Ashrafi et al., 2017)
2-Methyl-butanal	Aldehyde	Malty, pungent	*Staphylococcus aureus*	Bacterial culture studies	(Bos et al., 2013)
3-Methylbutanal	Aldehyde	Fruity, cheesy	*Proteus mirabilis*	Wound-derived samples (malignant breast cancer wounds) and bacterial culture studies	(Thuleau et al., 2018)
Hexanal	Aldehyde	Green, fatty, stale	Sterile autolysis (necrosis)	Necrosis/autolysis model	(Hoffman et al., 2009)
Heptanal	Aldehyde	Fatty, oily	Sterile autolysis (necrosis)	Necrosis/autolysis model	(Hoffman et al., 2009)
Nonanal	Aldehyde	Waxy, stale	Sterile autolysis (necrosis)	Necrosis/autolysis model and wound-derived samples	(Hoffman et al., 2009)
Methanol	Alcohol	Sharp, alcoholic	*Escherichia coli*, mixed flora	Bacterial culture studies	(Bos et al., 2013)
Pentanol	Alcohol	Sweet, alcoholic	*Escherichia coli*	Bacterial culture studies	(Bos et al., 2013)
3-Methyl-1-butanol	Alcohol	Alcoholic, fusel	AmpC-*E. coli strains*	Bacterial culture studies	(Dixon et al., 2022)
Acetoin	Ketone	Buttery, creamy	AmpC-*E. coli strains*	Bacterial culture studies	(Dixon et al., 2022)
Ethyl acetate	Ester	Solvent-like, overly sweet	*Escherichia coli*	Bacterial culture studies	(Bos et al., 2013; Dixon et al., 2022)
Pentyl ester	Ester	Fruity, banana/pear-like	*Candida albicans*	Fungal culture studies	(Costa et al., 2020; Fitzgerald et al., 2022)
Farnesol	Sesquiterpene	Sweet, floral, woody	*Candida albicans*	Fungal culture studies	(Costa et al., 2020; Fitzgerald et al., 2022)
Nerolidol	Sesquiterpene	Floral, woody	*Candida albicans*	Fungal culture studies	(Costa et al., 2020; Fitzgerald et al., 2022)
trans-Farnesol	Sesquiterpene	Sweet, woody	*Candida albicans*	Fungal culture studies	(Costa et al., 2020; Fitzgerald et al., 2022)
2,3-Dihydrofarnesol	Sesquiterpene	Floral, woody	*Candida albicans*	Fungal culture studies	(Costa et al., 2020; Fitzgerald et al., 2022)
Indole	Heterocycle	Fecal, animalic, overripe-sweet	*Escherichia coli*, anaerobes	Wound-derived samples (malignant breast cancer wounds) and bacterial culture studies	(Thuleau et al., 2018; Dixon et al., 2022)
Phenol	Aromatic compound	Medicinal, antiseptic-like	*Fusobacterium* spp.	Wound-derived samples (malignant breast cancer wounds) and bacterial culture studies	(Thuleau et al., 2018)
Cadaverine	Biogenic amine	Putrid, decaying flesh	Enterobacteriaceae, anaerobes	Bacterial culture studies	(Zamfir et al., 2025)
Putrescine	Biogenic amine	Acrid, rotten meat	Enterobacteriaceae, anaerobes	Bacterial culture studies	(Zamfir et al., 2025)
Ammonia	Inorganic volatile	Sharp, pungent	Necrosis; mixed flora	Bacterial culture studies and wound monitoring studies	(Azimzadeh et al., 2025)
Hydrogen cyanide	Inorganic volatile	Bitter, almond-like	*Pseudomonas aeruginosa*	Bacterial culture studies	(Azimzadeh et al., 2025)

*Evidence type refers to the primary experimental basis supporting the reported association, including wound-derived samples, bacterial culture studies, fungal culture studies, and necrosis/autolysis models.

A similarly complex volatilome is observed in chronic wound-associated pathogen *Escherichia coli* (*E. coli*). The known *E. coli* pan-volatilome comprises 66 metabolites, including characteristic malodorous ones like indole, ethyl acetate (solvent-like, overly sweet aroma), methanol (sharp, alcoholic), and pentanol (sweet, alcoholic) (Bos et al., 2013; Lin et al., 2021; Dixon et al., 2022).

Beyond species-specific signatures, VOC profiling can even resolve heterogeneity within a single pathogen. Dixon et al. (2022) demonstrated that AmpC-producing *E. coli* strains exhibit VOC profiles distinctively different from those of the susceptible strain. The AmpC-producing strain has five significantly increased VOCs: acetoin (buttery, creamy), 3-methyl-1-butanol (alcoholic), an unknown alkane, indole, and an unknown benzene derivative (Dixon et al., 2022). A similar degree of intra-species diversity is seen in *Klebsiella pneumoniae* (*K. pneumoniae*), where antibiotic stress reveals strain-specific odor patterns. Resistant strains emit a distinct VOC signature dominated by 3-methyl-1-butanol, thereby distinguishing them from susceptible strains (Li et al., 2024). These findings demonstrate that VOC profiles vary not only between bacterial species but also between strains of the same species.

Beyond more specific signatures, wound-associated bacteria often have overlapping yet characteristic VOC patterns, including ammonia (sharp, urine-like) and amines (rotten fish aroma), as well as ketones and alcohols such as ethanol (Salinas Alvarez et al., 2019). Among the dominant contributors to malodorous wounds are the biogenic diamines cadaverine and putrescine, produced by many wound-associated bacteria via amino acid decarboxylation (Michael, 2016). Their presence therefore reflects active bacterial colonization of necrotic wound tissue and is characteristic of wounds dominated by a variety of gram-negative rods and anaerobes, particularly Enterobacteriaceae such as *P. mirabilis*, *K. pneumoniae*, *Enterobacter* spp., and *Serratia marcescens* and *E. coli*. These compounds are described as having an intense acrid, putrid odor that lingers and may even induce nausea or vomiting (Zamfir et al., 2025).

*Candida albicans* (*C. albicans*), a pathogenic yeast present in approximately 22% of all chronic wounds, produces a distinct volatilome that differs markedly from that of bacterial pathogens (Kalan et al., 2016). It includes propionic acid, pentyl ester, and several sesquiterpenes: farnesol, nerolidol, trans-farnesol, and 2,3-dihydrofarnesol (**Table 1**). Sesquiterpenes are described as having a more pleasant, sweet, floral-like scent with woody undertones, while pentyl ester is known for its fruity, ripe banana- or pear-like aroma. However, when these compounds are mixed with rancid, sweaty, or body-odor–like volatiles such as propionic acid, the contrast between “sweet” and “rancid” notes can intensify the overall sensory impact, rendering the resulting smell even more repellent (Costa et al., 2020; Fitzgerald et al., 2022).

Even in the absence of microbial colonization, tissue undergoing necrosis emits a characteristic set of VOCs generated by sterile autolysis, including ammonia from amino-acid deamination and lipid-derived carbonyls such as hexanal, heptanal, and nonanal from membrane peroxidation. These compounds can produce faint sharp, “fatty,” or “stale” notes and reflect endogenous tissue breakdown rather than infection-driven malodor (Hoffman et al., 2009). However, humans exhibit substantial inter-individual variability in their VOC emission profiles.

### 3.2 Reported clinical strategies for the management of wound odor

Although wound odor is a common problem in chronic wound care, the available literature describes relatively few, non-standardized management strategies, which are largely supported by limited or low-level evidence. Reported approaches primarily reduce wound odor by lowering microbial burden or by physically absorbing or masking volatile compounds, none directly target wound odor perception at the level of the olfactory system. Across studies, the available evidence is heterogeneous and largely based on small studies with limited methodological standardization. Outcome measures vary widely across studies, ranging from subjective odor ratings to indirect clinical endpoints, limiting direct comparisons and robust assessment of efficacy. The following procedures are described in the literature.

#### 3.2.1 Odor control through suppression of bacterial load

Current clinical strategies primarily focus on reducing bacterial burden in the wound, with improvements in malodor emerging as a beneficial secondary effect rather than as a direct treatment target. In clinical practice, the topical antibiotic metronidazole is described as an effective off-label treatment to control wound odor, especially in palliative wound care and malignant tumor wounds (Castro and Santos, 2015; Villela-Castro et al., 2018). Metronidazole is particularly active against anaerobic bacteria, which are frequent contributors to malodor in chronic wounds. In a 2014 survey, 56.9% of respondents reported using metronidazole for infected wounds, and 87.9% rated its effect on odor as “somewhat effective” or “very effective” (Gethin et al., 2014). However, given the small sample size and observational nature of the data, the true effectiveness of metronidazole for odor control remains uncertain (Gethin et al., 2023).

Silver dressings are widely used as antimicrobial dressings in wound management and can indirectly reduce the odor by lowering the bacterial burden (Kalemikerakis et al., 2012). Although some studies report reductions in wound size, exudate, and odor compared with non-silver dressings in infected chronic wounds, most fail to provide robust comparative evidence using appropriate control groups (Bale et al., 2004; Beam, 2009).

Manuka honey-based wound dressings are described to reduce bacterial burden mainly by lowering the wound pH (Manuka honey pH=3.2-4.5) and methylglyoxal; in studies, this was associated with partial control of wound odor (Gethin and Cowman, 2005; Lund-Nielsen et al., 2011; Adderley and Holt, 2014; Drain and Fleming, 2015). There was some evidence that other sugar-based wound dressings could also reduce wound odor indirectly by inhibiting bacterial growth via osmosis (Chiwenga et al., 2009; Biswas et al., 2010; Murandu et al., 2011).

Iodine, commonly used as an antiseptic, disrupts bacterial cell walls, thereby reducing the microbial burden in the wound and potentially reducing wound odor (Akhmetova et al., 2016). The main drawbacks of iodine-based therapies are the sharp, unpleasant odor they emit and their comparatively high cytotoxicity (Hayashida and Yamakawa, 2021).

#### 3.2.2 Odor masking by pragmatic approaches

In addition to antimicrobial and antiseptic approaches, wound odor is frequently managed through pragmatic, symptom-oriented measures that aim to mask the malodor. In both clinical practice and home care, such strategies often resemble “home remedies” and are driven by the immediate need to render the odor more tolerable for patients, relatives, and caregivers (Gethin et al., 2014; Samala and Davis, 2015). One example is the use of wound dressings containing cinnamon, which mask malodor by generating a natural, spicy aroma (Ngô et al., 2025). Alternatively, coffee grounds are sometimes placed in the patients’ room to conceal wound odor (Samala and Davis, 2015). Aromatherapeutic approaches are also used, for example, by placing strong essential oils, such as peppermint oil, on the outside of the dressings, thereby overlaying malodor with a more pleasant scent (Mercier and Knevitt, 2005; Gethin et al., 2014; Samala and Davis, 2015).

#### 3.2.3 Odor reduction via VOC adsorption

A further group of interventions aims to reduce wound odor through physical removal of volatile compounds rather than through antimicrobial activity. Charcoal-based dressings exemplify this approach, as they are designed to adsorb malodorous VOCs directly from the wound environment (Seaman, 2006; Mikhalovsky et al., 2012). This effect is mediated by activated carbon, a charcoal derivative with a high binding capacity for volatile molecules, which has been shown to provide partial odor control in clinical settings (Hayashida and Yamakawa, 2021). Outside of formal wound care products, similar adsorption principles are applied pragmatically by placing cat litter in the patient’s room, where its high absorbent capacity sequesters malodorous volatiles from the surrounding air (Samala and Davis, 2015).

### 3.3 Deorphanization of olfactory receptors

#### 3.3.1 Deorphanization and antagonization of human olfactory receptors for wound-associated VOC

Given that current interventions act upstream at the level of microbial burden or volatile capture, the literature to date has not fully addressed the molecular interface at which odor is perceived: the olfactory receptor. Addressing wound odor at the level of perception requires deorphanization, i.e., linking individual VOCs to the olfactory receptors they activate, a task achieved through a variety of *in silico* and *in vitro* experimental deorphanization approaches. Viewed across methodologies, while the literature describes a diverse methodological landscape, the vast majority of successful human OR–ligand pairings over the past two decades are based on a single experimental core: heterologous expression in mammalian cells. Although *in silico* predictions and genomics-based OR-odorant mapping now function as integral upstream components of modern deorphanization pipelines by preselecting likely odorants or receptor–ligand candidates, thus reducing time and effort for wet-lab screening, functional validation of these interactions remains essential (Odoemelam et al., 2025). Native olfactory neuron–based systems retain the highest physiological fidelity, yet neither has displaced heterologous expression as a stand-alone deorphanization platform.

A growing set of human olfactory receptors responsive to VOCs associated with malodorous wounds have been identified through receptor deorphanization studies. However, the strength of evidence linking individual wound-associated VOCs to specific receptors varies considerably, ranging from direct human receptor assays and genetic association studies to heterologous expression systems, computational predictions, and non-human experimental models. Beyond receptor identification, antagonists have been described for several of these ORs. (**Table 2**) These compounds bind the receptor with little or no stimulatory activity, thereby reducing odor perception under experimental conditions.

**Table 2:** Overview of human receptors/detectors for wound-associated VOCs and known antagonists.

VOC (wound-relevant)	Primary receptor/detector	Evidence type	Known antagonist(s)	Key sources
Acetic acid	OR51E2	Human receptor assay	α-ionone; C80	(Saito et al., 2009; Pluznick et al., 2013; Wolf et al., 2017)
Propionic acid	OR51E2	Human receptor assay	α-ionone; C80	(Saito et al., 2009; Pluznick et al., 2013)
Isovaleric acid	OR51E1; OR11H7P	Human receptor assay; genetic association	2-ethylhexanoic acid (OR51E1)	(Menashe et al., 2007; Mainland et al., 2015; Jovancevic et al., 2017)
Butyric acid	OR51E1	Human receptor assay	2-ethylhexanoic acid	(Menashe et al., 2007; Mainland et al., 2015)
3-methyl-2-hexenoic acid (3M2H)	OR51B2	Human receptor assay; genetic association	Not established	(Li et al., 2022)
Dimethyl trisulfide (DMTS)	OR2T1/OR2T11	Computational prediction; indirect human receptor evidence	β-ionone	(Li et al., 2016; Fukutani et al., 2023)
Dimethyl disulfide (DMDS)	OR2T1/OR2T11	Computational prediction; indirect human receptor evidence	β-ionone	(Li et al., 2016; Fukutani et al., 2023)
Methanethiol (model thiol)	OR2T1/OR2T11	Human receptor assay	β-ionone	(Li et al., 2016; Fukutani et al., 2023)
2-/3-Methylbutanal	OR1A1; OR2W1	Human receptor assay	Not established	(Saito et al., 2009; Haag et al., 2022)
Hexanal/Heptanal/Nonanal	OR1A1; OR2W1; OR1G1	Human receptor assay	Not established	(Saito et al., 2009; Charlier et al., 2012; Haag et al., 2022)
Alcohols (ethanol, 3-methyl-1-butanol, pentanol)	Multiple low-specificity ORs	Human receptor assay	Not established	(Liu et al., 2025)
Indole	No definitive human OR	Mouse receptor studies	α-ionone; Z95; Hivernal^®^Neo; Lilyfore^®^	(Pfister et al., 2020)
Nerolidol	OR2B3; OR2M4	Human receptor assay	Not established	(Gonzalez-Kristeller et al., 2015)
Ammonia	TRPV1/TRPA1	Mouse receptor studies	Not established	(Fujita et al., 2008; Dhaka et al., 2009)
Trimethylamine (TMA)	TAAR5	Human receptor assay; human sensory studies	Timberol^®^	(Wallrabenstein et al., 2013; Wallrabenstein et al., 2015)
Putrescine/Cadaverine	TAAR6/TAAR8 (predicted)	Computational prediction	Not established	(Izquierdo et al., 2018)

Short-chain fatty acids, which generate sour, rancid, “vomit-like” and “cheese-like” notes in malodorous wounds, converge on a small ensemble of human olfactory receptors. Acetic and propionic acids are among the most robustly characterized ligands for human OR51E2, as they fit the receptor’s unusually small ligand-binding pocket, which sterically excludes longer chains (Saito et al., 2009; Pluznick et al., 2013; Pronin and Slepak, 2021). Importantly, OR51E2 is not only highly specific but also antagonizable: α-ionone acts as a competitive antagonist, and more recently, intracellular allosteric inhibitors, such as C80, have been identified (Wolf et al., 2017; Abaffy et al., 2024). Additionally, isovaleric acid, propionic acid and butyric acid are detected by human OR51E1 *in vitro* experiments (Mainland et al., 2015; Pronin and Slepak, 2021), whose activation can be antagonized by 2-ethylhexanoic acid (Jovancevic et al., 2017). Genetic and functional studies identify OR11H7P, an OR for recognition of “sweaty odors, as a principal receptor for isovaleric acid and a key determinant of individual sensitivity. Individuals carrying two disrupted alleles are largely insensitive, whereas those with at least one intact allele exhibit markedly increased detection (“hyperosmia”) (Menashe et al., 2007). Closely related receptors within the same subfamily, including OR11H4 and OR11H6, also showed responsiveness in receptor assays, consistent with the principle that ORs within a subfamily bind chemically similar ligands with sensitivity (Menashe et al., 2007). Genetic and functional studies indicate that OR51B2 contributes to human perception of the axillary odorant 3-methyl-2-hexenoic acid (3M2H), with genetic variation in this receptor determining individual sensitivity to this compound (Li et al., 2022).

Sulphur-containing VOCs such as DMTS and DMDS are among the most potent contributors to chronic wound malodor and are perceived as intensely pungent. In human heterologous expression systems, members of the human OR2T receptor family, particularly OR2T11 and OR2T1, are highly sensitive to low–molecular–weight thiols such as methanethiol and related sulfur compounds (Li et al., 2016; Fukutani et al., 2023). Although direct human receptor data for DMTS and DMDS remain limited, their structural and chemical similarities to experimentally validated OR2T ligands suggests that OR2T-family receptors may contribute to the perception of sulphur-containing wound VOCs. However, direct receptor validation for these specific compounds remain incomplete. Fukutani et al. (2022) identified β-ionone as an antagonist of OR2T1 and OR2T11, consistent with human sensory studies demonstrating reduced perception of sulphurous odors in the presence of β-ionone (Fukutani et al., 2023).

The wound-associated aldehydes 2-Methyl-butanal, 3-methylbutanal, heptanal, nonanal, and hexanal do not produce a single, sharply defined odor note but instead contribute to a diffuse malodor background. Rather than engaging a dedicated receptor, these aldehydes have been shown in heterologous human OR assays to activate multiple broadly tuned ORs, most prominently OR1A1 and OR2W1, thereby likely contributing to the overall olfactory “noise” of the wound environment rather than a discrete percept (Sanz et al., 2005; Schmiedeberg et al., 2007; Saito et al., 2009; Cometto-Muñiz and Abraham, 2010; Haag et al., 2022). Moreover, nonanal exhibits high affinity for another aldehyde-binding OR, OR1G1 (Charlier et al., 2012). A comparable pattern is observed for wound-associated alcohols such as ethanol, methanol, 3-methyl-1-butanol, and pentanol, which appear to engage weakly with multiple receptors with low specificity (Liu et al., 2025).

A dedicated human receptor for indole, a prominent VOC produced by *E. coli* and other wound-associated microorganisms, has not yet been conclusively identified, but several indole-responsive mouse ORs are known. Using these models, Breheny et al. identified α-ionone as a strong antagonist across all indole-responsive mouse ORs, while Pfister et al. demonstrated that compounds such as Z95, Hivernal^®^Neo, and Lilyfore^®^ partially suppressed perception of the pungent, fecal-like indole odor (Pfister et al., 2020). Whether these approaches translate to indole perception in humans remains to be established and will require direct experimental validation in human sensory studies.

Fungal volatiles, mainly produced by *C. albicans*, intensify wound malodor by adding “sweet” and “woody” notes to the otherwise pungent olfactory profile (Fitzgerald et al., 2022). The human olfactory receptors, OR2B3 and OR2M4, were deorphanized for nerolidol, whereas no specific human ORs have yet been identified for the other wound-associated sesquiterpenes farnesol, trans-Farnesol, and 2,3-Dihydrofarnesol (Gonzalez-Kristeller et al., 2015).

Beyond classical olfactory receptors, humans possess additional chemosensory systems for volatile detection. Ammonia, for example, is primarily detected by a distinct class of sensors: nociceptive ion channels, particularly TRPV1 and TRPA1, which respond to chemically reactive and potentially toxic volatiles (Fujita et al., 2008; Dhaka et al., 2009). These channels function as direct danger sensors rather than as part of normal olfactory coding. The hydrogen cyanide (HCN) produced by *P. aeruginosa* has been proposed as a potential ligand for TRPA1 based on the channel’s responsiveness to chemically related irritants, including tear gas compounds such as CN, CS (Brône et al., 2008). Activation of these pathways likely contributes to the sharp, stinging, and aversive character of certain wound odors.

Biogenic amines, such as putrescine and cadaverine, often referred to as the “odors of death,” are detected by trace-amine-associated receptors (TAARs) in humans, which are GPCRs closely related to ORs. Computational models have indicated TAAR6 and TAAR8 as candidate sensors for cadaverine and putrescine (Izquierdo et al., 2018), however, to date, only one human TAAR has been conclusively deorphanized. TAAR5 has been identified as a receptor for trimethylamine (TMA), and experimental studies have shown that Timberol^®^ can antagonize this receptor, resulting in reduced perception of the characteristic “fishy odor” associated with trimethylamine (Wallrabenstein et al., 2013; Wallrabenstein et al., 2015).

#### 3.3.2 Linking human olfactory perception to interindividual differences

Genomics-based OR–percept mapping provided an interesting additional angle on wound odor perception, identifying receptors that contribute to the human perception of a given odor by linking genetic variation in OR genes to interindividual differences in perceptual phenotypes, effectively connecting receptor genotype to sensory experience (Trimmer et al., 2019). Humans perceive odors differently, not least because of substantial interindividual differences within the OR family. This was exemplified by March et al. (2018), who demonstrated that genetic variation in OR7D4, an androstenone-responsive receptor, is associated with marked differences in odor perception. Individuals carrying the wild-type variant typically described androstenone as sickening, foul, or urine-like, whereas carriers of the WM variant, which renders the receptor largely unresponsive, tend to perceive the odor as more pleasant (March et al., 2018).

## 4 Discussion

Wound odor is a multifactorial problem that burdens not only patients but also relatives and caregivers, and is frequently ranked by patients as one of the most distressing aspects of their condition (Stuermer et al., 2025). In addition, chronic wounds comprise a heterogeneous group of conditions, including diabetic foot ulcers, venous leg ulcers, pressure injuries, and malignant wounds, which may differ in microbial composition, tissue pathology, and VOC production. A central challenge in wound odor management lies in the complexity and heterogeneity of its origins, which vary significantly between individuals. In infected wounds, odor is largely shaped by the colonizing microbiota, with key pathogens in chronic wounds including *S. aureus*, *P. aeruginosa*, and Enterobacteriaceae, making characteristic contributions to the olfactory profile through the associated VOCs such as DMTS, DMDS, indole, a variety of aldehydes, and more (Shirasu et al., 2009; Bos et al., 2013; Salinas Alvarez et al., 2019). However, it is not only the bacterial presence that is relevant, but also their relative abundance, which determines the intensity, quantity, and quality of the odor. In parallel, the extent of bacteria-mediated and vascular-related necrosis in and around the wound modifies the odor profile significantly, as the by-products of these processes, such as ammonia, putrescine, and cadaverine, are perceived as particularly aversive (Hoffman et al., 2009; Michael, 2016; Zamfir et al., 2025). It is therefore crucial not only to determine which VOCs are present in wounds, but also to identify those that are most irritating and distressing to humans, enabling their selective targeting by therapeutic strategies.

Wound odor is not driven by abundance alone; available evidence suggests that some VOCs may disproportionately influence perception through strong receptor activation, whereas others contribute primarily to a more diffuse olfactory background (Launay et al., 2012; Achebouche et al., 2022). This hierarchy suggests that future odor-targeted interventions should focus on the few high-impact odorants that actually drive distress, rather than attempting to neutralize all VOCs present in the wound. Genomics-based OR–percept mapping does not capture wound odor in its full complexity, but it provides crucial evidence that for certain odorants, human perception is dominated by a single receptor with strong genetic effects (Trimmer et al., 2019). Studies on OR11H7P and isovaleric acid, or OR7D4 and androstenone, show that interindividual differences in distress can hinge on one molecular interface (Menashe et al., 2007; March et al., 2018; Trimmer et al., 2019). In the context of wound odor, this suggests that not all VOCs contribute equally to the patient’s and HCP’s experience, and that a small number of receptor–ligand pairs may disproportionately shape what is perceived as most offensive.

While a number of studies have begun to link wound-associated VOCs to their perceptual qualities, substantial gaps remain in identifying and functionally characterizing the key odor-driving compounds. A major limitation is that bacterial odor profiles are often generated outside their physiological context, for example, by culturing isolated bacterial strains on agar and analyzing emitted VOCs. Such approaches neglect the complex biochemical and ecological conditions of real wounds and may misrepresent both composition and sensory relevance. This gap between analytical VOC profiling and the actual sensory experience of wound odor has direct clinical consequences. Without a clear, perception-oriented understanding of which compounds truly drive distress, wound-odor management remains largely trial-and-error (Gethin et al., 2014; Akhmetova et al., 2016; Gethin et al., 2023).

An additional limitation in wound care, and one that is particularly pronounced in odor-targeting interventions, is the lack of standardized, large-scale, controlled studies evaluating such interventions. Reported benefits of current approaches are often derived from small case series, uncontrolled designs, and heterogeneous outcome measures, resulting in findings that are difficult to compare, reproduce, or verify. Moreover, many established interventions act indirectly: agents such as metronidazole, silver-based products, sugar, or Manuka honey are primarily used to reduce microbial load, with any associated reduction in wound odor occurring as a secondary effect of their antibacterial activity (Beam, 2009; Murandu et al., 2011; Kalemikerakis et al., 2012; Adderley and Holt, 2014; Gethin et al., 2014; Gethin et al., 2023). Other wound dressings or adjuvant measures aim not to reduce odor at its source, but to mask or absorb it, for example, through “household remedies” such as coffee or cinnamon, or by using adsorptive materials such as activated charcoal (Mikhalovsky et al., 2012; Samala and Davis, 2015; Hayashida and Yamakawa, 2021). An additional emerging strategy is the modulation of the wound microbiome itself. Rather than targeting individual VOCs or their perception, microbiome-based therapies aim to alter the microbial community responsible for VOC production. Recent approaches include genetically engineered lactic acid bacteria designed to deliver therapeutic proteins directly within the wound environment, thereby promoting tissue repair, angiogenesis, and immune modulation. Preclinical studies have demonstrated improved wound healing following topical application of engineered Lactococcus lactis, while first-in-human investigations using genetically modified Limosilactobacillus reuteri expressing CXCL12 have reported favorable safety profiles and signals of enhanced wound healing (Kurkipuro et al., 2022; Öhnstedt et al., 2023). Although these approaches were developed primarily to accelerate wound closure rather than reduce malodor, successful modulation of wound microbiota and the wound microenvironment could theoretically reduce the production of malodor-associated VOCs by altering microbial community composition and metabolic activity within the wound. At present, however, their effects on wound odor remain largely unexplored and require dedicated investigation. Accordingly these approaches act upstream or peripherally, and leave the sensory processing of wound odor in the human nose unaddressed. Nevertheless, olfactory receptors represents a potentially promising point of intervention. In principle, novel VOC-specific strategies could act at the level of olfactory perception by reducing receptor activation by malodorous compounds in the nasal cavity and thereby attenuating odor perception. Conceptually, olfactory receptor antagonization may be viewed as a form of perceptual silencing, in which receptor activation by malodorous VOCs is reduced rather than the odor being masked by added fragrances or removed by absorbent materials. Collectively, these limitations highlight that current wound odor management strategies primarily address the chemical source of odor, whereas sensory mechanisms underlying odor perception remain largely unexplored as therapeutic targets.

Recent advances in VOC characterization and olfactory receptor deorphanization have enabled the identification of olfactory receptors for several wound-associated VOCs, although the strength of evidence varies considerably between receptor–ligand pairs. Human OR51E1, for example, detects SCFAs such as isovaleric, propionic, and butyric acid (Mainland et al., 2015; Pronin and Slepak, 2021), while broadly tuned receptors including human OR1A1 and OR2W1 respond to wound-associated aldehydes such as 2- and 3-Methylbutanal, nonanal, heptanal, and hexanal (Saito et al., 2009; Cometto-Muñiz and Abraham, 2010; Haag et al., 2022). For other key odorants, receptor engagement is currently supported primarily by computational and structure-based predictions. Dimethyl sulfides (DMTS, DMDs), for instance, have been proposed as candidate ligands for low-molecular-weight thiol receptors such as OR2T11 and OR2T1 based primarily on computational and structure-based modelling studies (Li et al., 2016; Fukutani et al., 2023). These interactions, however, still require functional confirmation in heterologous expression systems. Identifying these receptors does more than explain how malodor is perceived, it may ultimately provide the opportunity to intervene at the sensory interface.

Recent advances, particularly the integration of *in silico* modelling and AI-based prediction frameworks, have substantially improved the efficiency of OR deorphanization by enabling a more targeted and scalable approach to receptor-ligand discovery. Rather than screening more than 400 human ORs indiscriminately, computational preselection now enables focused experimental testing of the most plausible receptor-ligand pairs. While functional validation remains indispensable, this hybrid pipeline substantially accelerates progress and enables systematic mapping of wound-relevant VOCs to their receptors (Launay et al., 2012; Yasi et al., 2019; Yasi et al., 2020; Achebouche et al., 2022; Odoemelam et al., 2025). Heterologous expression models in human cell lines have enabled the deorphanization of many human ORs through relatively straightforward processes. However, a fundamental limitation of this approach remains its reduced physiological fidelity (Zhuang and Matsunami, 2008; Ieki et al., 2022) with ORs being expressed outside their native cellular context (Bush and Hall, 2008; Peterlin et al., 2014). This limitation contrasts with native olfactory sensory neuron approaches, often regarded as the “gold standard” of deorphanization, which better reflect physiological reality but are technically demanding, low-throughput, and almost exclusively rodent-based. While human and rodent OR repertoires are broadly conserved, one-to-one orthology is difficult to resolve, and ligand specificity is not reliably preserved (Peterlin et al., 2014). As a result, receptor-ligand pairings established in rodents cannot be assumed to translate directly to human olfaction. For example, human OR2J2 responds strongly to the medium-chain alcohol 1-octanol, whereas its rodent orthologs show weaker and shifted activation profiles (Adipietro et al., 2012), illustrating that even closely related receptors can diverge functionally across species. Taken together, current evidence suggests that robust OR deorphanization is best achieved through an integrative strategy combining computational prediction, rodent OSN data, and human heterologous validation, thereby balancing biological relevance with experimental feasibility.

Against this background, deorphanization is relevant not only for identifying agonists of wound-relevant VOCs but also for revealing antagonists, e.g., molecules that block receptor activation, demonstrating that olfactory signaling can be modulated pharmacologically at the receptor level (Pfister et al., 2020). In the context of malodor, this opens a potential therapeutic dimension: rather than removing or masking distressing VOCs, perception itself may be modulated through antagonizing the receptors involved in odor detection. This is particularly attractive for “key” receptors that dominate perception of specific malodorous compounds, where modulation of a single receptor-ligand interaction could disproportionally influence the perception of a major odor component. The human receptor for short-chain fatty acids, OR51E1, which detects isovaleric, propionic, and butyric acids, is antagonizable by 2-ethylhexanoic acid (Mainland et al., 2015; Jovancevic et al., 2017), providing a mechanistically defined example of receptor-level odor suppression that may warrant exploration in a clinical context. Likewise, for the sulfur-sensitive receptors OR2T1 and OR2T11, β-ionone has been identified as a functional antagonist that attenuates sulphurous perception (Fukutani et al., 2023). Whether this antagonism extends to the clinically dominant bacterial odorants DMTS and DMDS remains to be tested, but it is a promising translational hypothesis. At the same time, broadly tuned receptors such as OR1A1 or OR2W1 represent strategic targets, as they respond to entire classes of wound-associated aldehydes and related VOCs. In principle, antagonists targeting such broadly tuned receptors could represent an attractive strategy for modulating the perception of multiple wound-associated aldehydes and related VOCs simultaneously (Schmiedeberg et al., 2007; Saito et al., 2009; Haag et al., 2022). However, this concept remains hypothetical and has not yet been evaluated in the context of wound malodor.

In practice, effective management of malodorous wounds is unlikely to be achieved through a single receptor antagonist. Because multiple VOC classes contribute to the overall odor, effective suppression will probably require a combination of antagonists targeting a few key receptors. Moreover, translating receptor antagonism into a clinical therapy raises practical and safety challenges. The optimal mode of delivery remains open, room sprays, nasal sprays, or dressings releasing antagonists from a non-contact top layer are all conceivable, but differ in spatial reach, duration of action, and patient acceptance. Importantly, potential off-target perceptual effects must be considered, particularly when targeting broadly tuned receptors, such as OR1A1 or OR2W1, which have been implicated in the perception of multiple structurally diverse odorants, including many encountered in everyday life.

Several limitations of the current evidence base should be acknowledged. Many VOC studies rely on isolated microbial cultures and may therefore not fully reflect the complexity of the wound volatilome (Bos et al., 2013; Kalan et al., 2016; Ashrafi et al., 2017; Liegenfeld et al., 2025). In addition, the strength of evidence supporting individual receptor–VOC assignments varies substantially, ranging from direct human receptor studies to computational predictions and non-human models (Menashe et al., 2007; Saito et al., 2009; Mainland et al., 2015). Finally, olfactory receptor antagonization remains a largely preclinical concept, and clinical studies demonstrating meaningful reductions in wound malodor are currently lacking (Pfister et al., 2020; Fukutani et al., 2023; Abaffy et al., 2024). Therefore, receptor-targeted odor management should presently be regarded as a promising translational framework rather than an established therapeutic approach.

## 5 Conclusion

Advances in VOC characterization and olfactory receptor research have substantially improved our understanding of odor perception, but further studies are needed to determine which VOCs in real wounds truly drive malodor. Studies in patients and translational skin models could provide information currently missing from classic *in vitro* assays using isolated bacterial cultures. Most importantly, future research should focus on identifying the wound-associated VOCs, that contribute most strongly to distress in patients, relatives, and caregivers. This requires integrating human perception into VOC analysis. One promising approach could be to combine gas chromatography with sniffing ports, allowing individual VOC fractions derived from wound samples (e.g., dressings) to be smelled and ranked by perceived repulsiveness. Such perception-guided approaches may help prioritize clinically relevant odors rather than merely the most abundant. Future progress will require a systematic pipeline that links wound-derived VOCs to their human olfactory receptors under clinically relevant conditions. Together with continued advances in receptor deorphanization and antagonist discovery, these approaches may ultimately enable the development of odor-targeted interventions that complement existing wound care strategies. By addressing malodor at the level of perception, rather than solely at the level of microbial burden or volatile production, a new therapeutic dimension for wound odor management may emerge, with the potential to improve quality of life for patients and those involved in their care.

## Data Availability

The original contributions presented in the study are included in the article/**Supplementary Material**. Further inquiries can be directed to the corresponding author.
