# Supplementary material for: Microbial volatile organic compounds and olfactory receptors in wound malodor
Source: Front Cell Infect Microbiol. 2026 Jul 10;16:1833939. doi: 10.3389/fcimb.2026.1833939 (PMC13395670; doi:10.3389/fcimb.2026.1833939)
Supplement: Supplementary file 1 [file DataSheet1.pdf]

Supplementary Material

Supplementary Table S1. Database-specific search strategies.

Database	Search strategy	Additional filters
MEDLINE (via PubMed)	"bacterial volatile organic compounds"	Publications 2000–2025
MEDLINE (via PubMed)	"volatile organic compounds" AND "wound"	Publications 2000–2025
MEDLINE (via PubMed)	"wound odor" OR "wound odour"	Publications 2000–2025
MEDLINE (via PubMed)	"malodorous wounds"	Publications 2000–2025
MEDLINE (via PubMed)	"human olfactory receptors"	Publications 2000–2025
MEDLINE (via PubMed)	"deorphanization" AND "olfactory receptor"	Publications 2000–2025
ScienceDirect	"wound odor" AND NOT "plants" AND NOT "food"	Publications 2000–2025; Research Articles and Review Articles only
ScienceDirect	"bacterial volatile organic compounds"	Publications 2000–2025; Research Articles and Review Articles only
ScienceDirect	"malodorous wounds"	Publications 2000–2025; Research Articles and Review Articles only
ScienceDirect	"human olfactory receptors"	Publications 2000–2025; Research Articles and Review Articles only
ScienceDirect	"deorphanization" AND "olfactory receptor"	Publications 2000–2025; Research Articles and Review Articles only
ScienceDirect	(wounds AND human) AND "volatile organic compounds" AND NOT plants AND NOT food	Publications 2000–2025; Research Articles and Review Articles only
Cochrane Library	"bacterial volatile organic compounds"	Publications 2000–2025
Cochrane Library	"volatile organic compounds" AND "wound"	Publications 2000–2025
Cochrane Library	"wound odor"	Publications 2000–2025
Cochrane Library	"malodorous wounds"	Publications 2000–2025
Cochrane Library	"human olfactory receptors"	Publications 2000–2025
Cochrane Library	"deorphanization" AND "olfactory receptor"	Publications 2000–2025

Note: Searches were performed as separate concept-based queries because the review addressed multiple interdisciplinary topics, including wound malodor, bacterial VOCs, olfactory receptors, and receptor deorphanization. Relevant records retrieved from the different search concepts were subsequently screened according to predefined eligibility criteria.
